# Ultrasonic Lateral Displacement Sensor for Health Monitoring in Seismically Isolated Buildings

**DOI:** 10.3390/s150717000

**Published:** 2015-07-13

**Authors:** Iwao Matsuya, Fumiya Matsumoto, Ikuo Ihara

**Affiliations:** Department of Mechanical Engineering, Nagaoka University of Technology, Kamitomioka 1603-1, Nagaoka 940-2188, Niigata, Japan; E-Mails: fumy1991@gmail.com (F.M.); ihara@mech.nagaokaut.ac.jp (I.I.)

**Keywords:** structural health monitoring, earthquake, seismically isolated building, lateral displacement, air-coupled ultrasound, sound field, normal distribution, polynomial approximation

## Abstract

An ultrasonic lateral displacement sensor utilizing air-coupled ultrasound transducers is proposed. The normally-distributed far field of an ultrasound transducer in a lateral direction is taken advantage of for measuring lateral displacement. The measurement system is composed of several air-coupled ultrasound transducers as a receiver and several transmitters. The transmitters are immobilized at a fixed point, whereas the receiver set-up is separately arranged on the opposite side. In order to improve measurement accuracy, a correction method that utilizes polynomial approximation is introduced. The difference between the corrected lateral displacement and the reference displacement is estimated to be 0.2 mm at maximum for the two transmitters system. A good responsiveness is demonstrated by conducting a dynamic response experiment. When five transmitters are arranged, their measurement range is easily extended up to ±60 mm with an accuracy of 0.7 mm. In both cases, the fluctuations to the measurement ranges show less than 1%. These results indicate that the developed sensor system is useful for measuring relative lateral displacement of a seismically isolated building in the field of structural health monitoring.

## 1. Introduction

**Figure 1:**
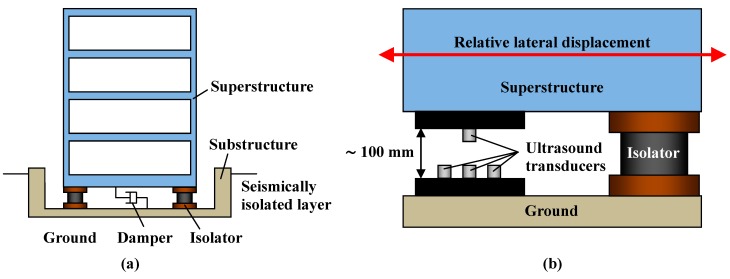
(**a**) Seismically isolated building; (**b**) Enlarged view of the seismically isolated layer and installation of ultrasonic lateral displacement sensor.

Since the late 1990s, the number of seismically isolated buildings has been increasing with the purpose of preventing structural damage and ensuring safety in case of earthquakes [1,2,3,4,5,6,7,8]. If a large earthquake strikes a seismically isolated building, an isolated layer will absorb the bulk of the seismic motion energy leading to drastically different results when compared to a conventional building. Figure 1a shows a seismically isolated building. In this building, the seismically isolated layer is arranged between the ground and the first floor. The isolated layer consists of dampers and isolators that support the superstructure. The isolators are made of laminated rubber and work like a spring which allows a large horizontal displacement of the superstructure. The dampers reduce the displacement response of the superstructure. Although the movement of a conventional building with several stories has been measured at approximately 2 Hz during an earthquake, the dominant response of a seismically isolated building is reduced to around 0.1 to 0.5 Hz, which is similar to the displacement response of a high-rise building. The natural frequency of a seismically isolated building is generally determined by the damping ratio of the isolated layer. In addition, a large displacement between the superstructure and the ground, which is estimated to be approximately 200–300 mm for a relatively severe earthquake, is generated in the seismically isolated building. These characteristics of seismically isolated buildings reduce damage to structural/non-structural members and allow them to maintain their functionality after an earthquake. In the field of structural health monitoring (SHM), a significant amount of research about measuring the acceleration of the motion response of buildings has been reported [9,10,11,12,13,14], but direct measurements of the lateral displacement of seismically isolated buildings in real time are particularly valuable. If the lateral displacement of the isolated layer can be measured directly in real time, the deterioration of the laminated isolated rubber can then be evaluated appropriately. However, the maximum lateral displacement of the isolated layer of a seismically isolated building is extremely wide, and it varies case-by-case according to the characteristics of the isolator. Furthermore, the distance between the superstructure and the ground seems to vary in different buildings, depending on their design. Finally, the lateral displacement of the layer returns to the origin at the end of an earthquake, so we cannot evaluate the damage related to the maximum displacement. These factors have made the measurement of the lateral displacement of the isolated layer difficult for conventional sensors.

The accelerometer based SHM technique has been developed for measuring the movement of individual stories in a building during an earthquake [13,14]. CCD (charge-coupled device) video camera-based displacement monitoring techniques have also been investigated [15]. Sharp needles have been utilized to draw the lateral motion of seismically isolated layer [7]. However, the method of monitoring displacement derived from the double integration of acceleration cannot track residual displacement that is related to the movement of the building at very low frequency [16]. The CCD-based technique demands a very complex processing procedure and computing equipment, which reduce the cost performance of the system [17,18]. Moreover, if the distance between the upper and lower floor is very short and the required displacement range is very wide, which are considered representative traits of the seismically isolated layer, the CCD-based measurement system requires a specially designed lens such as a fisheye lens. The sharp needles cannot be utilized in real-time monitoring and lack reliability. Therefore, a simple, alternative monitoring method for relative lateral displacement inside a seismically isolated building is strongly sought. For this purpose, a lateral displacement sensor with a wide measurement range possessing a relatively short distance between the upper and the lower sensor installation spots, particularly one less than 100 mm, is demanded.

In this paper, an ultrasonic lateral displacement sensor using air-coupled ultrasound transducers is proposed. Figure 1b shows an enlarged view of the isolated layer and the installation of the displacement sensor. Its measurement system is composed of several air-coupled ultrasound transducers as a receiver and several transmitters. First, we will show the measurement principal and theory for measuring lateral displacement using the characteristics of the air-coupled ultrasound transducer. Second, real time monitoring of lateral displacement is shown. Then, how to extend the measurement range is described. From the viewpoints of measurement accuracy and dynamic response, the feasibility of the proposed method in structural health monitoring is discussed.

## 2. Experimental Setup

### 2.1. Ultrasound Intensity Distribution of Air-Coupled Ultrasound Transducer

**Figure 2:**
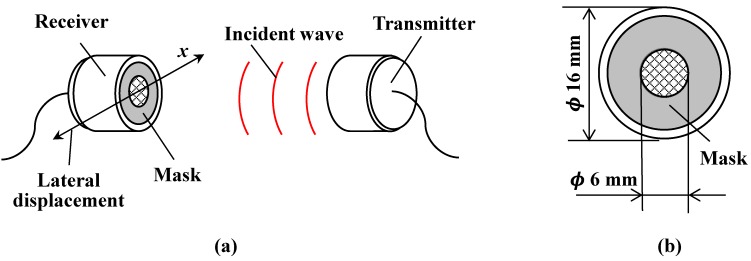
(**a**) Experimental setup for measuring ultrasound intensity distribution of air-coupled ultrasound transducer; (**b**) Front view of the receiver.

In order to measure the relative lateral displacement over a wide range, the characteristics of the air-coupled ultrasound are utilized. The intensity of the propagating ultrasound waves in the far field follows a Gaussian distribution in a lateral direction [19,20]. Before measuring lateral displacement, the ultrasound intensity distribution of the air-coupled ultrasound transducer must be characterized. Figure 2 shows the experimental setup for measuring the intensity distribution using the relative lateral displacement of a pair of transducers. As shown in Figure 2a, a transmitter and a receiver of air-coupled ultrasound transducers (UT/UR1612MPR, *ϕ*16 mm in diameter, 40 kHz for central frequency, SPL Limited, Hong Kong) are arranged at a distance of 60 mm and face each other. The installation space is usually very narrow in seismically isolated buildings, it is essential to show capability of measuring lateral displacement over shorter distances. The distance between transducers is determined by the power of the ultrasonic transducer. So if another ultrasonic transducer is utilized, the distance will be spread more easily. However, we have selected an ultrasonic transducer with lower power in order to measure the displacement over as short of a distance as possible. Because the intensity of the ultrasound beam fluctuates extremely in the near field, which is approximately 14.2 mm, the far field of the transducer is utilized in this experiment [20]. A mask with a central hole is attached to the receiver as shown in Figure 2b. The receiver should receive ultrasound waves only in its central part, which makes the shape of the ultrasound intensity distribution more clear. This mask is made of heavy paper and blocks ultrasound waves at the outer edges of the receiver. The diameter of the mask aperture is designed to be 6 mm, so it can obtain a normally-distributed ultrasound intensity. The origin corresponds to the center of the transmitter. The transmitter is driven by a pulse signal from a square-wave pulsar with a repetition rate of 40 kHz. When the ultrasonic waves are received, the ultrasonic signals are converted into voltage signals inside the receiver. The voltage signals are captured using a 12 bit serial acquisition board at a sampling rate of 100 MHz. The bit level of the sampling is approximately 2.4 mV. In order to reduce the background noise of the signals, the bandpass filter from 30 to 50 kHz is utilized and the average values are calculated from thirty acquired signals. The maximum voltage value is extracted and recorded as the ultrasound intensity. When the receiver is displaced parallel to the *x* direction from the origin, the ultrasound intensity plots of propagating ultrasound waves are measured according to the *x* position of the transmitter.

### 2.2. Measurement Principal for Lateral Displacement Sensing

**Figure 3:**
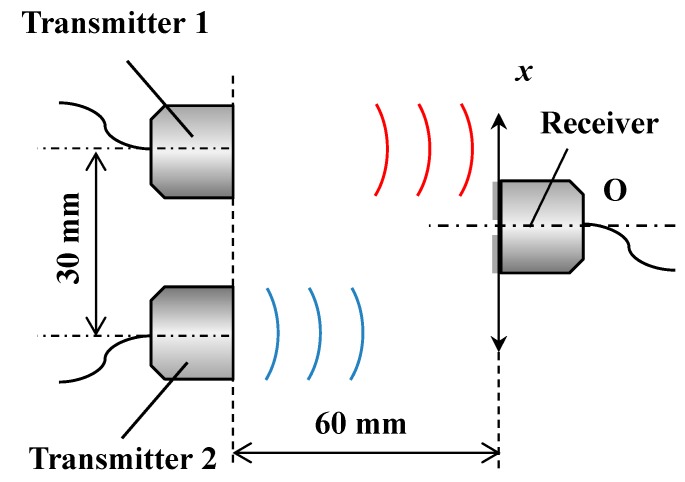
Experimental setup for measuring relative lateral displacement sensing using two transmitters.

Figure 3 shows the experimental setup used for relative lateral displacement sensing. The measurement system is composed of two air-coupled ultrasound transducers as transmitters and the same transducer also used as a receiver. Two transmitters are immobilized at a fixed point side by side with a distance of 30 mm, whereas a receiver is separately arranged on the opposite side. The distance between the transmitters and the receiver is set at 60 mm. The origin is set to be the center of the transmitter 2. When the receiver is displaced parallel to the *x* direction from the origin, two ultrasound intensity distributions are measured by the receiver according to the *x* position of the receiver. As a static displacement test, the receiver on the *X*-stage is displaced ±15 mm and the measurement accuracy is evaluated. The required specification for the fluctuation value depends on the percentage of the length of each measurement range. For the health monitoring of a seismically isolated building, it is thought that if the fluctuation rate is less than 1% in each measurement range, a high-precision measurement will be successfully carried out. Next, a dynamic response experiment is conducted to verify the accuracy and capability to follow the dynamic movement in real-time. The receiver is moved ±15 mm with around 0.6 Hz in the dynamic response experiment. This movement of the stage simulates the relative movement of the superstructure in the seismically isolated building.

### 2.3. Measuring Lateral Displacement over a Wide Measurement Range

**Figure 4:**
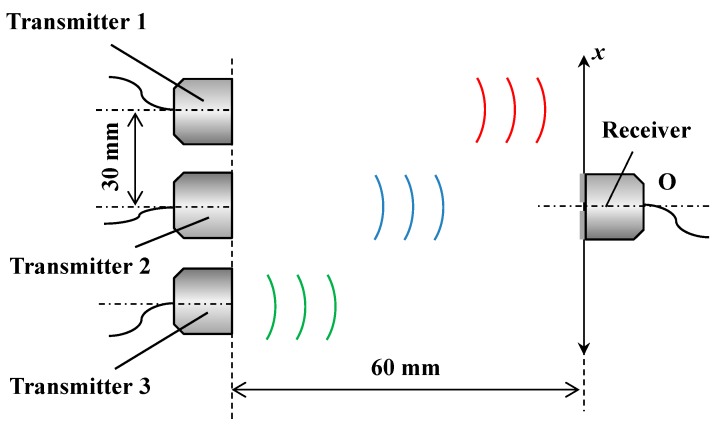
Experimental setup for measuring lateral displacement over a wide range.

In SHM, real-time responsiveness is important for small displacements as mentioned above, but for larger displacements, recording the maximum displacement is essential. Therefore, the ability to extend for large lateral displacements should be shown. The measurement range of lateral displacement is easily extended utilizing several transmitters and a receiver. Figure 4 shows the experimental setup for measuring lateral displacement over a wide measurement range. Several transmitters are immobilized at a fixed point, side by side with a distance of 30 mm, whereas the receiver is separately arranged on the opposite side. The distance between the transmitters and receiver is arranged to be 60 mm. When the receiver is traveling along the *x* axis, the wider lateral displacement of the receiver is calculated during every section of every two transmitters. The accuracy of the lateral displacement over a wide measurement range is discussed.

## 3. Results and Discussion

### 3.1. Ultrasound Intensity Distribution of Air-Coupled Ultrasound Transducer

**Figure 5:**
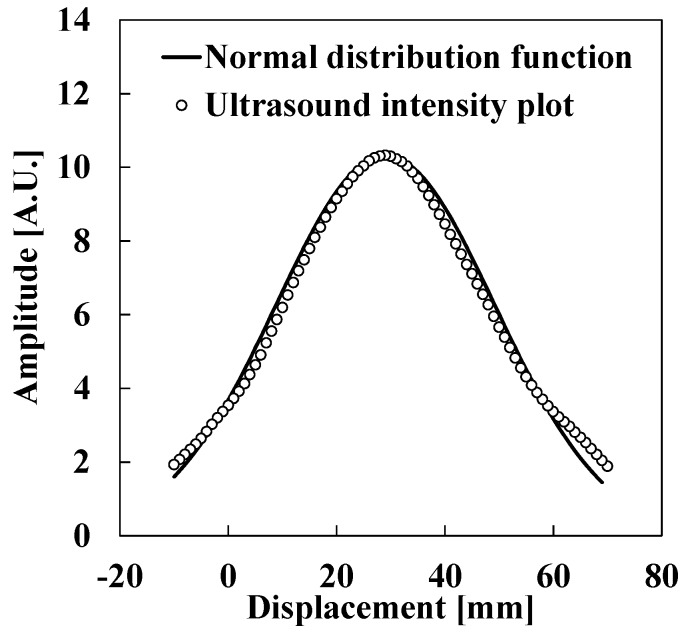
Normally distributed ultrasound intensity of the air-coupled ultrasound transducer.

Figure 5 shows the ultrasound intensity distribution of the air-coupled ultrasound transducer by conducting the experiment shown in Figure 2. In Figure 5, open circles show the ultrasound intensity as measured by the receiver in each position. The solid line shows the fitted Gaussian curve. It is clear that these plots can be approximated by the Gaussian curve fairly well. The fitted curve is expressed using the following normal distribution function:

(1) $$f(x)=\frac{1}{\sqrt{2\pi \sigma^{2}}}\exp\left[-\frac{{\left(x-\mu \right)}^{2}}{2\sigma^{2}}\right]$$

where *x* is the relative lateral displacement of the receiver from its origin, *f*(*x*) represents the standardized ultrasound intensity of the received ultrasound, *σ* is the standard deviation of the data plots, and *μ* is the position of the transmitter. In the following section, we will describe the measurement method for lateral displacement utilizing the characteristics of the air-coupled ultrasound transducer.

### 3.2. Measurement Principal for Lateral Displacement Sensing

**Figure 6:**
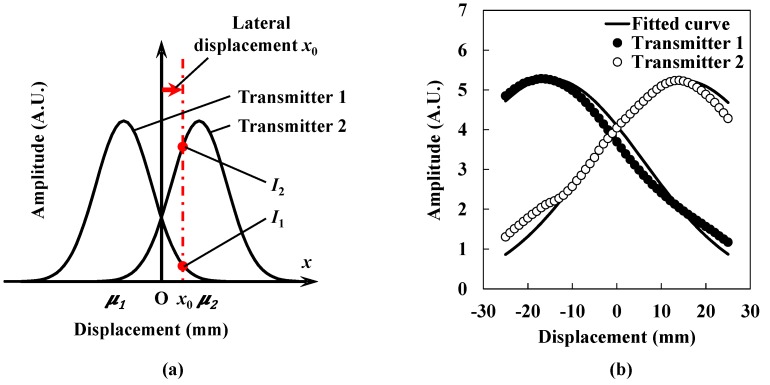
(**a**) Schematic of two ultrasound intensity distributions; (**b**) Ultrasound intensity distributions were measured by the receiver according to its lateral displacement.

Figure 6a shows the schematic of two ultrasound intensity distributions obtained by carrying out the experiment shown in Figure 3. When the receiver is displaced laterally in front of the transmitters, the data plots from two transmitters are represented through the utilization of the above mentioned Gaussian approximation as follows:

(2) $$I_{1}=\frac{1}{\sqrt{2\pi {\sigma_{0}}^{2}}}\exp\left[-\frac{{\left(x_{0}-\mu_{1}\right)}^{2}}{2{\sigma_{0}}^{2}}\right]$$

(3) $$I_{2}=\frac{1}{\sqrt{2\pi {\sigma_{0}}^{2}}}\exp\left[-\frac{{\left(x_{0}-\mu_{2}\right)}^{2}}{2{\sigma_{0}}^{2}}\right]$$

(4) $$\mu_{1}+\mu_{2}=d$$

where *I*_1_ and *I*_2_ show the ultrasound intensity transmitted by transmitters 1 and 2, respectively, *x*_0_ is the relative lateral displacement of the receiver from the origin, *σ*_0_ is the standard deviation of the ultrasound intensity distribution which is common to both transmitters, *μ*_1_ and *μ*_2_ are the original positions of the transmitters in *x* direction, and *d* is the interval between transmitters 1 and 2. Note that in the experiment detailed in this paper, the ultrasound transducers are alternately turned on and off, so each ultrasound beam is generated as an independent event. From Equations (2–4), the relative lateral displacement *x*_0_ is derived:

(5) $$x_{0}=A\ln\left(\frac{I_{1}}{I_{2}}\right)+B$$

(6) $$A=-\frac{{\sigma_{0}}^{2}}{d}$$

(7) $$B=\frac{d}{2}$$

Equation (5) indicates that the lateral displacement *x*_0_ is expressed as a logarithm of the ratio of *I*_1_ to *I*_2_, which are obtained from the two transmitters. If the calibration for the displacement measurement is carried out, we can obtain the values of *A* and *B* as known constants beforehand in Equations (6) and (7). Even if the shapes of the data curves obtained from transmitters 1 and 2 are not identical to each other, the difference in the standard deviations and their absolute values are included by the constants *A* and *B* as long as the Gaussian fitting is applied to *I*_1_ and *I*_2_. Therefore, the lateral displacement *x*_0_ is successfully derived by measuring the logarithm of the ratio of *I*_1_ to *I*_2_. If a wider range of lateral displacement measurement is required, the arrangement of continuous pairs of ultrasound transducers will make it possible. Figure 6b shows the actual data plots representing the ultrasound intensity distributions according to the position of the receiver. The open circles and closed circles show the ultrasound intensities obtained from transmitters 1 and 2, respectively. The solid line shows the fitted curve that is explained after the following procedure.

**Figure 7:**
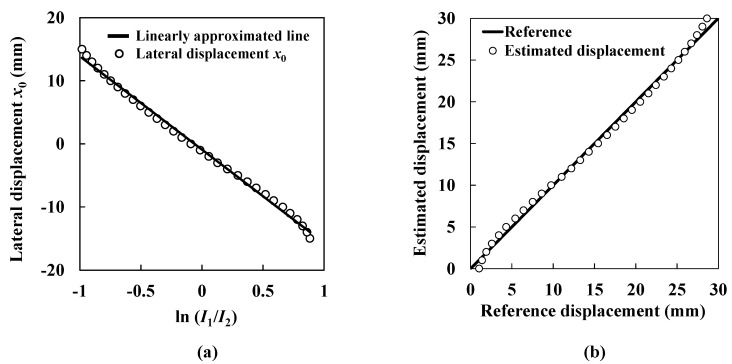
Estimation of lateral displacement. (**a**) Lateral displacement and the logarithm of the ratio of the ultrasound intensities obtained from the two transmitters; (**b**) Estimated lateral displacement.

Figure 7a shows the data plots of lateral displacement according to the logarithm of the ratio of the ultrasound intensities obtained from the two transmitters. The solid line shows a linearly approximated line that is expressed as follows:

(8) $$x_{0}=-14.79\ln\left(\frac{I_{1}}{I_{2}}\right)-0.89$$

Constants *A* and *B* are obtained by Equation (8). As described, relative lateral displacement *x*_0_ is obtained. In Figure 7b, the fitted curve is also drawn by using Equations (5), (6) and (8). Figure 7b shows the estimated lateral displacement compared with the reference displacement. The open circles show the estimated lateral displacement. The solid line shows the reference displacement. Although the estimated lateral displacement basically agrees with the reference, it fluctuates considerably and its maximum value is 1.36 mm. As mentioned above, we have tried to obtain more accurate ultrasound intensity distributions by utilizing a mask on the receiver. However, as shown in Figure 4, the ultrasound intensity distribution still remains imperfect, which causes fluctuations in the calculated lateral displacement. Therefore, it is considered that a further correction method is essential for obtaining more precise lateral displacement measurements for health monitoring of seismically isolated buildings.

**Figure 8:**
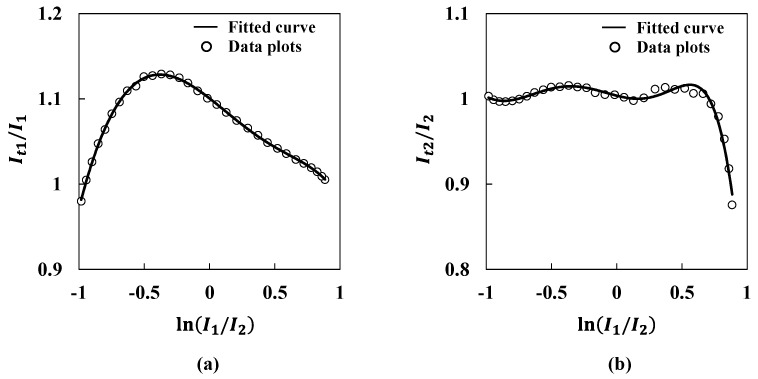
Derivation of compensation coefficients (**a**) *e*_1_ and (**b**) *e*_2_.

**Figure 9:**
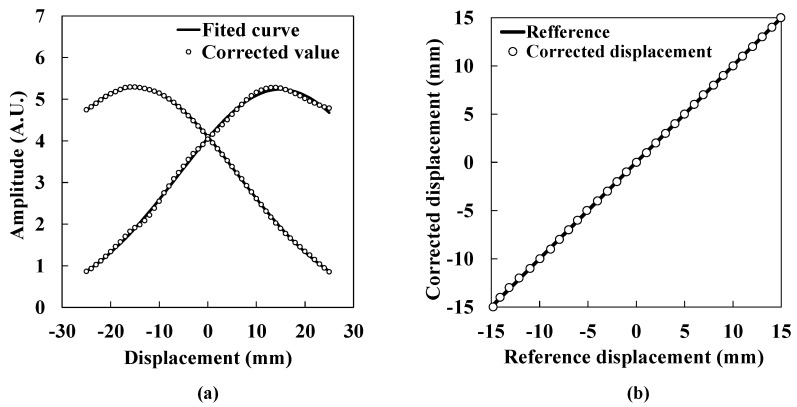
(**a**) Corrected ultrasound intensity distribution; (**b**) Corrected lateral displacement.

There is a non-negligible gap between obtained ultrasound intensity distributions and fitted curves that are based on Gaussian approximations as shown in Figure 6b. This gap seems to cause a fluctuation in calculated lateral displacement. So, we introduce a compensation coefficient to the gap as follows:

(9) $$I_{t1}=e_{1}\left(\ln\left(\frac{I_{1}}{I_{2}}\right)\right)\cdot I_{1}$$

(10) $$I_{t2}=e_{2}\left(\ln\left(\frac{I_{1}}{I_{2}}\right)\right)\cdot I_{2}$$

where *I*_t1_ and *I*_t2_ are normally approximated values that are described on the fitted curve as shown in Figure 6b, *e*_1_ and *e*_2_ are compensation coefficients for the obtained ultrasound intensities *I*_1_ and *I*_2_, respectively. Compensation coefficients are expressed as functions of the logarithm of the ratio of *I*_1_ to *I*_2_ in Equations (9) and (10). Figure 8 shows the values of *I*_t1_/*I*_1_ and *I*_t2_/*I*_2_ by changing the logarithm of the ratio of *I*_1_ to *I*_2_ to derive the compensation coefficients *e*_1_ and *e*_2_. For these coefficients, polynomial approximation is applied because the values of *I*_t1_/*I*_1_ and *I*_t2_/*I*_2_ fluctuate greatly as shown in Figure 8. The compensation coefficients are represented as follows:

(11) $$I_{t1}/I_{1}=e_{1}\left(\ln\left(\frac{I_{1}}{I_{2}}\right)\right)=-0.26y^{6}-0.04y^{5}-0.04y^{4}+0.16y^{3}-0.05y^{2}+0.12y-1.10$$

(12) $$I_{t2}/I_{2}=e_{2}\left(\ln\left(\frac{I_{1}}{I_{2}}\right)\right)=-0.15y^{6}-0.37y^{5}-0.06y^{4}+0.26y^{3}-0.06y^{2}+0.04y+1.00$$

where *y* shows the logarithm of the ratio of *I*_1_ to *I*_2_. As shown in Figure 8 and Equations (11) and (12), the compensation coefficients were successfully obtained. Figure 9a shows the corrected ultrasound intensity distribution with the application of Equations (11) and (12). Open circles show corrected values. The solid line shows the fitted curve previously shown in Figure 6b. The fitted curve and the corrected values overlap fairly well. Figure 9b shows the corrected lateral displacement. Open circles show the corrected lateral displacement. The solid line shows the reference displacement. From the Figures, it is clear that the corrected lateral displacement agrees well with the reference. The fluctuation between corrected values and the reference is within 0.20 mm at maximum. Because the fluctuation rate for the 30 mm range corresponds to 0.67%, it is verified that this system has sufficient accuracy for a small displacement region. For the actual measurement activity, a manual stage needs to be arranged under the transmitters or the receiver for the system calibration. After the calibration, the stage should be fixed as a base keeping its position at the origin.

**Figure 10:**
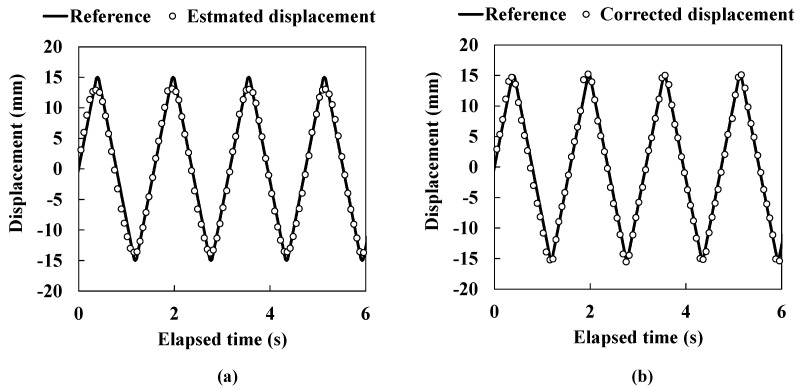
The dynamic response experiment in 0.6 Hz movement. (**a**) Estimated lateral displacement; (**b**) Corrected lateral displacement.

Figure 10 shows the results of the dynamic response experiment. The solid line shows the reference displacement and the open circles show the estimated displacement in Figure 10a as well as the corrected displacement in Figure 10b. In both Figure 10a,b, a good capability for the dynamic response can be seen. By using the correction method, as expected, better accuracy was achieved compared to methods utilizing only Gaussian distributions as shown in Figure 10a,b. Generally, a building shakes with high frequency over a small displacement region compared rather than a large displacement region. Therefore, our sensor system required a high responsiveness performance appropriate for small displacement regions. However, the sensor application in this paper is applied to a seismically isolated building that is shaken with several Hz at most. Additionally, the ultrasound propagates at around 340 m/s, which is much faster than the movement of the seismically isolated layer. Because the distance between transducers is set to be very close in the sensor application, it is considered that this sensor follows the seismic motion well over both small and large displacement regions.

### 3.3. Measuring Lateral Displacement over a Wide Range

**Figure 11:**
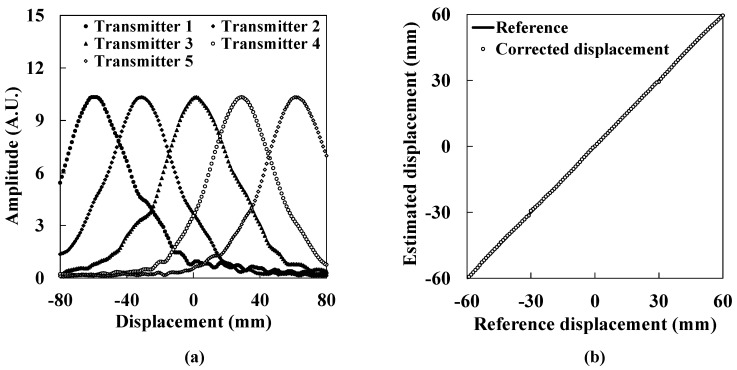
Measuring lateral displacement over a wide range. (**a**) The ultrasound intensity distribution obtained from five transmitters; (**b**) The corrected lateral displacement.

Figure 11 shows the experimental results for the measurement of lateral displacement over a wide range using several ultrasound transmitters. Figure 11a shows the ultrasound intensity distributions obtained from five transmitters. Figure 11b shows the corrected lateral displacement. The open circles show the corrected lateral displacement. The solid line shows the reference displacement. The corrected lateral displacement agrees well with the reference within a 0.70 mm fluctuation. Because the fluctuation rate for the 120 mm range is found to be 0.58%, it is verified that this system has sufficient accuracy for large displacement regions. In addition, this result indicates that the measurement range is easily extended using several air-coupled ultrasound transducers. As mentioned above, the maximum lateral displacement of the isolated layer varies case-by-case according to the damping ratio of the isolator. Therefore, it is important for this sensor to be able to extend its measurement range without limitation when installing it in seismically isolated buildings.

## 4. Conclusions

A new method of measuring the relative lateral displacement inside a seismically isolated building directly for the monitoring of structural health is demonstrated. In this method, the far field of an ultrasound transducer is employed to avoid fluctuations of the ultrasound beam. The normally distributed ultrasound intensity distribution of the air-coupled ultrasound is utilized for obtaining relative lateral displacement between transmitters and the receiver. In order to improve measurement accuracy, compensation coefficients derived using the polynomial approximation, which corrects the obtained ultrasound intensity distribution to the ideal Gaussian distribution, are introduced. The difference between the corrected value and the true value is estimated to be within 0.2 mm (0.66% fluctuation) for the two transmitter system and 0.7 mm (0.58% fluctuation) for the five transmitter system. Both results indicate that the sensor system has sufficient accuracy because both fluctuation rates are less than 1%. A good responsiveness was demonstrated by conducting a dynamic response experiment. If several transducers are arranged, the measurement area will be extended wider or even become two-dimensional according to the number of transducers. The wider distance between the transmitters and receiver will be achieved using higher output power ultrasound transducer in the future. These results indicate that the developed measurement system is useful for the health diagnosis of structures.
